# 

**Published:** 1918-01-26

**Authors:** 


### CONTENTS.

PAGE

**Consumptive Combatants** ... ... ... ... 345

**The Belgian Doctors and Pharmacists** ... ... 346

**Hospital and Institutional News** ... ... ... 347

Hospital Treatment of Discharged Soldiers—Captain Tunnard's Successor—Restricted Hotel Meals—Honour to a Medical Hero—How to Reduce the Number of Vacant Military Beds—The Need for a New Policy—Fewer Board Meetings—The Value of Hospital Visitors—The Leeds Medical School—A Graceful Gift—The Finances of Edinburgh Royal Infirmary—The Power of the Pen—The Disabled Soldier in Bristol—Birmingham Children's Hospital—Tribunals and Tuberculosis—Rations in Institutions—The Latest Report on the Poor Law—The *Searchlight*—This Week's Drug Market.

**Lord Lister** ... ... ... ... ... ... ... 351

His Work, His Teaching, and Benefits to Mankind.

**Leicester Royal Infirmary** ... ... ... ... 352

The Year's Work and War Prices.

**The War and Orthopædics** ... ... ... ... 353

How to Secure Efficient Surgery.

**The Prevention of Scurvy** ... ... ... ... 354

Some Recent Researches upon Vitamines.

**Preparing for Air Raids** ... ... ... ... ... 355

The Organisation in North Lambeth.

**Hospital Architecture and Construction** ... 356

The Royal Lancaster Infirmary (Temporary Wing).

PAGE

**The Editor's Letter-Box** ... ... ... ... 356

"London" Nurses and their Emoluments.

The Medico-Psychological Association.

**The Growth of the Nursing Profession** ... 357

II. Become Founders and Helpers of Democratic Government.

**Nursing Progress and its Developments** ... 357

VI. The Probationer's First Two Years.

Round the Hospitals.

Mr. Smallwood's Speech—Did the Matron Act on Instructions?—The late Miss Thorold—Where Nurses' Associations have failed—Activity of Poor-Law Matrons—Jam-Making Secure in 1918.

**Some Problems of To-Day** ... ... ... ... 361

New Powers for Urban and District Councils.

**Matrons' and Sisters' Appointments** ... ... 362

**The Book Market** ... ... ... ... ... ... 362

**Parliament and the Profession** ... ... ... 362

**Church Collections for the Hospital Sunday Fund** ... ... ... ... ... ... ... 362

**The Institutional Worker** ... ... (Suppl.) 1

Stiff Joints and Other Injuries.

Enquiries and Answers.

The Sick and in Need.

War Matters.

Employment and Training.

Miscellaneous.

